# Pharmaceutical nanotechnology: from the bench to the market

**DOI:** 10.1186/s43094-022-00400-0

**Published:** 2022-01-15

**Authors:** Zaed M. Mazayen, Amira M. Ghoneim, Rasha S. Elbatanony, Emad B. Basalious, Ehab R. Bendas

**Affiliations:** 1 Faculty of Pharmacy, Department of Pharmaceutical Technology, Future University in Egypt, Cairo, 11835 Egypt; 2 Faculty of Pharmacy, Department of Pharmaceutics and Industrial Pharmacy, Cairo University, Cairo, Egypt

**Keywords:** Nanotechnology, Delivery systems, Fabrication, Characterization, Applications

## Abstract

**Background:**

Nanotechnology is considered a new and rapidly emerging area in the pharmaceutical and medicinal field. Nanoparticles, as drug delivery systems, impart several advantages concerning improved efficacy as well as reduced adverse drug reactions.

**Main body:**

Different types of nanosystems have been fabricated including carbon nanotubes, paramagnetic nanoparticles, dendrimers, nanoemulsions, etc. Physicochemical properties of the starting materials and the selected method of preparation play a significant aspect in determining the shape and characteristics of the developed nanoparticles. Dispersion of preformed polymers, coacervation, polymerization, nano-spray drying and supercritical fluid technology are among the most extensively used techniques for the preparation of nanocarriers. Particle size, surface charge, surface hydrophobicity and drug release are the main factors affecting nanoparticles physical stability and biological performance of the incorporated drug. In clinical practice, many nanodrugs have been used for both diagnostic and therapeutic applications and are being investigated for various indications in clinical trials. Nanoparticles are used for the cure of kidney diseases, tuberculosis, skin conditions, Alzheimer’s disease, different types of cancer as well as preparation of COVID-19 vaccines.

**Conclusion:**

In this review, we will confer the advantages, types, methods of preparation, characterization methods and some of the applications of nano-systems.

## Background

Nanotechnology is the molecular-scale fabrication of various functioning systems. These systems have special physical, electrical, and optical characteristics that make them appealing in a variety of domains, ranging from materials science to biology [1]. Nanomedicine is one of the most well-known nanotechnology research fields. It uses nanotechnology to develop highly targeted medicinal interventions for disease detection, prevention, and treatment [2]. Over the last few decades, there has been a spike in nanomedicine research, which is currently being turned into commercialization activities around the world, culminating in the marketing of numerous products. Drug delivery systems now dominate nanomedicine, with revenues accounting for over 75% of total sales [3]. Nanoparticles have a diameter of 10–1000 nm. Entrapped, encapsulated, dissolved, or linked to the nanoparticle matrix is the active pharmaceutical ingredient (API) [4]. Nanoparticles can be made by altering the method of fabrication. Nanoparticles have been proven to be useful as drug delivery vehicles. Many uses for nanoparticulate drug delivery systems exist, including gene therapy, cancer therapy, AIDS therapy, and radiation. It can also be used to transport proteins, antibiotics, and vaccinations, as well as serve as vesicles to cross the blood–brain [5]. The major aims of nanoparticle design as a delivery system are to control particle size, surface properties, and drug delivery and API release so as to ensure site-targeted drug activity at an appropriate therapeutic rate and dosing regimen. In this review, we will discuss the advantages of nanoparticles as drug delivery systems, different types of nanosystems and their applications. We will also explain different methods used in the fabrication and characterization of nanoparticles. Examples of marketed nanoparticles products will be provided as well.

## Main text

### Benefits of utilizing nanoparticles as a drug delivery system

The benefits of employing nanoparticles as drug vehicles are because of two key characteristics: their tiny size and the use of biodegradable materials in the majority of cases [6, 7]. The effectiveness of most medication delivery methods is found to be largely reliant on particle size. Drug nanoparticles exhibit increased solubility and superior bioavailability which is a result of their small particle size and large surface area [8]. Additionally, their ability to cross the blood brain barrier, entering pulmonary system, endothelium of tumors and absorption through tight junctions of skin endothelial cells, give them added value. The nano-range size of these particles, in general, allows for effective absorption by various cell types as well as selective drug accumulation in the target locations [9, 10].

Nanoparticles also have the benefit of being more adequate for intravenous administration than conventional microparticles. The smallest body capillaries have a diameter of 5–6 m. To make sure that particles do not cause embolism, the size of particles dispersed in the circulation should be substantially less than 5 m [11]. Using both natural and synthetic biodegradable polymers for nanoparticle preparation give them the advantages of targeted drug delivery, improve bioavailability and achieve sustained release behavior of medications from a single dose at the target site over a prolonged period of time; by adaptation of the system, endogenous enzymes can be prevented from destroying the drug [12].

Furthermore, typical oral or injectable medicines now accessible for use are not necessarily provided in the most suitable formulation. As a result, goods containing proteins or nucleic acids will require more creative carrier systems (nanoparticles) to improve their efficacy and avoid any instability [13].

### Types of pharmaceutical nanosystems

#### Carbon nanotubes

Carbon nanotubes were first found in 1991 [14]. They are tubular carbon-based structures. These tubes are made up of cylinders of graphite sheets that are sealed at one or both ends by bucky balls and range in length from 1 to 100 nm. Single-walled nanotubes (SWNTs) and multiwalled nanotubes (MWNTs) are two designs that have recently gained popularity (MWNTs). C60-fullerenes are also found in typical configurations. They come in a variety of graphite cylinder configurations and are known for being cage-like and hollow (nanotubes and fullerenes). They are suitable for drug encapsulation because of their surface features and size, and they have crucial physical qualities. The DNA helix has a diameter of half the size of SWNTs. MWNTs, on the other hand, have diameters that range from a few nanometers to tens of nanometers, depending on how many walls they have in their structure [15]. Chemical vapor deposition, combustion procedures and electric arc discharge are the most common methods for producing fullerenes and carbon nanotubes. The strength and stability of these structures are used to characterize them so that they can be used as trustworthy drug transporters. Endocytosis or insertion across the cell membrane is how nanotubes enter cells. The structures of fullerenes were able exhibit tissue targeting and intracellular targeting of mitochondria. Additionally, it was found that that they show both antioxidant and antimicrobial activity [16].

#### Quantum dots

Quantum dots (QDs) are made up of semi-conducting structures that are 2–10 nm in size. They are nanocrystals with an inorganic semi-conductor core (CdSe) and an organic shell coated with zinc sulphide to increase optical qualities, and they are designed to glow when under the influence of light. The addition of a cap improves the solubility of QDs in aqueous buffers [17]. The particle's radius spans from 2 to 10 nm. Several qualities have been attributed to the long-term tracking of intracellular processes, bio-imaging in vitro, and real-time monitoring. Narrow emission, strong photo-stability, broad UV excitation, and brilliant fluorescence are some of these properties [18]. Cell labelling, biomolecule detection and biological performance, DNA hybridization, immunoassays, and the creation of non-viral vectors for gene therapy, carriers for cancer treatment, and transport vehicles for biological and non-biological agents are some of the diagnostic and therapeutic applications of QDs [19].

#### Nanoshells

Nanoshells are altered models for drug targeting, with a silica core and outer layer of metal [20]. Nowadays, much attention was gained by these nanoshells. The characteristics of these particles can be changed by adjusting the ratio between the core and the shell. It is now feasible to formulate these nanostructures in targeted physical properties, like size and morphology. Because all of the materials cannot be formulated in the desired morphologies, nanoshells are used to create new systems with a variety of morphologies. Particles of specific shapes could be covered with thin shell to achieve appropriate morphology. These shells have the advantage of being inexpensive since precious materials can be added to low-cost cores. As a result, precious material is needed in smaller quantities during synthesizing nanoshells [21]. Nanoshells targeting can be obtained by immunological techniques; an example for this targeting strategy is the gold nanoshells which were occupied with antibodies moieties on its outer gold surface for enhancement of the targeting power toward the cancer cell [22]. Nanoshells occupy different functions in varied areas like chemically stabilizing colloids, improving luminescence features and drug [23].

#### Nanobubbles

Nanobubbles are bubble-shaped particles formed at the nanoscale at the interface of lipophilic surfaces in liquids. When heated to body temperature, they mix to form microbubbles, which are stable at room temperature. They arise in supersaturated solutions as a result of gas nucleation at the hydrophobic surface, resulting in air gas trapping. There are 4 types of these nanoparticles; plasmonic, bulk, oscillating and interfacial nanobubbles. Drugs for cancer treatment were successfully loaded into these particles, they were able to target tumor tissues and increase the tumor cells uptake with the influence of ultrasound exposure [24, 25].

#### Paramagnetic nanoparticles

Magnetic nanoparticles are small particles with a diameter of fewer than 100 nm that can be controlled by a magnetic field. Magnetic elements are used to make these particle materials. The magnetic sensitivity of these nanoparticles is used to classify them. Magnetic susceptibility of paramagnetic nanoparticles is higher than that of typical contrast forms. These nanoparticles are utilized for diagnostic and treatment strategies. Paramagnetic nanoparticles targeting is effective for identification of specific organs [26].

#### Liposomes

Liposomes are synthetics particles made from amphiphilic phospholipids that self-assemble. They are made up of spherical double layered vesicles that surround an aqueous core domain that can range in size from 50 nm to several micrometres depending on the kind. General biocompatibility and biodegradability are appealing biological characteristics of liposomes. Liposomes are the most often utilized nanosystems as drug vehicles in clinical trials. They can be utilized to decrease medication clearance as well as reduce systemic effects and toxicity [27]. For the transfer of DNA, siRNA, proteins, and cancer treatments, nanoscale modified liposomes have good pharmacokinetic characteristics.

Low loading capacity, rapid drug release and the lack of adjustable drug release patterns are all limitations of liposomes [28]. Drugs are also discharged into the extracellular fluid because liposomes are unable to penetrate cells [29]. Following oral or parenteral administration, stability and structural integrity against a hostile bio-environment can be achieved by surface modification [30]. Drugs can be incorporated in the water phase of liposomes using an ammonium sulphate gradient to counteract the rapid release of the drug from liposomes. This will allow for consistent drug trapping and minimal drug loss throughout circulation [31]. Liposomes have also been coupled to antibodies to deliver medication to particular targets [32].

#### Niosomes

Niosomes are a type of molecular cluster formed in an aqueous phase by the self-assembly of non-ionic surfactants. Niosomes have a unique architecture that allows them to function as a new delivery method that can accommodate both lipophobic and lipophilic agents [33]. Niosomes consist of non-ionic surfactants, they are characterized by their non-toxicity, high stability and they are considered to be a replacement to liposomes. In vivo, niosomes act like liposomes; the entrapped drug's circulation is extended, and organ distribution and metabolic stability are changed. Characteristics of niosomes rely upon the bilayer, besides the preparation technique. It is proved that the entrapment volume during formulation decreases as a result of intercalation of cholesterol in the bilayers, and this leads to a reduction in the entrapment efficiency [34]. Present conclusions for the adoption of niosomes in the delivery of drugs are with a broad extent in entrapment of potent drugs [35], anticancer [36] and anti-viral medications [34].

#### Dendrimers

Dendrimers are a special category of polymers, characterized by being multi-branched, with a controllable size and shape. The size of these dendrimers is determined by the degree of branching, which may be regulated. Additionally, spherical branching within dendrimers creates voids that can be benefited for drug entrapment and delivery. The free ends of dendrimers, on the other hand, can be changed for conjugation to other molecules [37]. These nanostructures are advanced in terms of surface functionalization and stability, making them unique drug delivery possibilities. There are three main fundamental areas in terms of construction: core, branches, and surface. These networks help in the delivery of bioactives like medicines, genes, and vaccinations to specific tissues. Solubilization, gene therapy [38], dendrimer-based drug delivery [39], immunoassay, and MRI contrast agent are only a few of the uses for dendrimers.

#### Polymeric micelles

Polymeric micelles are a type of micelle made up of lipophilic and lipophobic monomer units in a block copolymer. They are made up of a center of lipophilic blocks that is stabilized by a corona of lipophilic polymeric chains. Corona-forming PEG blocks are utilized, and the length of a lipophilic center-forming block is similar to that of a hydrophilic block [40]. As a medication carrier, a micellar system has various advantages over conventional systems. Using micelle-forming surfactants to promote drug solubility improves the solubility of a weakly water-soluble medicine. They also increase the permeability of medications across physiological barriers, which improves their bioavailability. As a result, alterations in drug biodistribution occur. They help reduce negative side effects of critical medications. As a result of their reduced size and lipophilic shell, polymeric micelles remain in the blood for prolonged time after intravenous delivery, reducing their uptake by the reticulo-endothelial system. Micelles can also be made target specific by chemically attaching a targeting component to their surface. Because it is in a micellar form, the medication is effectively shielded from possible deterioration due to biological surroundings [41]. Because it is in a micellar form, it will find its path to the target organ or tissue.

#### Polymeric nanoparticles

Polymeric nanoparticles (PNPs) are mostly biodegradable and biocompatible; therefore, researchers are drawn to biodegradable PNPs as a drug-delivery system [42, 43]. PNPs are subdivided into vesicular systems (nanocapsules) and matrix systems (nanospheres). Advance modification of natural polymers was recently explored by researchers which comprises synthetic polyesters. One of the most familiar natural polymers is chitosan. Many polymers reduce toxic issues with the artificial polymers [44]. Natural PNPs prevailed over traditional delivery systems, due to their higher efficiency and effectiveness. Nevertheless, they have some drawbacks like poor reproducibility, degradation problems and potential antigenicity. The encapsulated drug's release behavior is controlled by the manufacturing technique. PNPs are potential intracellular and site-targeting systems.

#### Nanocapsules

Nanocapsules and nanospheres vary in that the former are carriers where the drug is contained in a core enclosed by a polymeric membrane, while the latter are structures where the drug is disseminated through the polymeric matrix [45]. PNPs can be thought of as a matrix in which the drug is equally distributed. Across or inside the polymeric matrix, the medication might be dissolved, entrapped, or encapsulated. PNPs are an excellent alternative for cancer therapy and other applications due to their capacity to customize medication delivery [46].

#### Solid lipid nanoparticles

Solid lipid NPs (SLN) were produced as a substitute to emulsions, liposomes and PNPs as a colloidal drug delivery system in a controlled manner [46]. SLNs are prepared from solid lipids and stabilized by surfactant(s). SLN offers several benefits for drug delivery over other particle carriers, including superior tolerability, biodegradability, high bioavailability via the ocular route, and a targeted impact on the brain [47, 48]. The research of SLN has exploded in recent years, notably with the high-pressure homogenization technique. SLN has been produced and studied for a variety of applications. The small size of SLN allowed them to become injected intravenously and used for site-targeting of drugs.

#### Nanoemulsions

Nanoemulsions and self-emulsified drug delivery systems (SEDDS) have gained a lot of interest in recent years as a way to increase the bioavailability of medicines of low aqueous solubility. Nanoemulsions are non-homogenous systems made up of immiscible liquids where one is disseminated as droplets in the other [49]. When integrated into aqueous phases under mild mixing, SNEDDS are isotropic mixes of oil, surfactant, co-surfactant, and drug that produce oil-in-water (o/w) nanoemulsions [50]. By a variety of processes these systems improve the oral bioavailability of weakly water soluble medicines. Furthermore, the small size of the droplets reduces the surface tension between the oil droplets and the aqueous medium of the gastrointestinal tract, allowing for more uniform and widespread drug distribution in the gut [51].

### Fabrication of nanoparticles

The physicochemical characteristics of the polymer as well as the selected drug determine the suitable method for the preparation. Nanoparticles mainly have been synthesized by different methods including dispersion of pre-formulated polymers, co-acervation of hydrophilic polymers and polymerization of monomers [52]. Other techniques have been mentioned in the literature for production of nanoparticles including supercritical fluid technology [53] and particle replication in non-wetting templates [54].

#### Dispersion of preformed polymers

This method relies on the fabrication of biodegradable nanoparticles through the dispersion of biodegradable polymers [55–57]. Dispersion of preformed polymers can be utilized in different ways:

##### Solvent evaporation method

One of the most commonly used procedures for the production of nanoparticles is solvent evaporation. Emulsification of the polymer solution in an aqueous phase is the initial stage, followed by evaporation of the polymer solvent, which results in polymer precipitation as nanospheres. This technique is dependent on the polymer's solubility as well as the hydrophobicity of the organic solvent. To produce an oil in water (o/w) emulsion, the drug-polymer combination is emulsified in an aqueous solution including a surfactant or emulsifying agent. The organic solvent is then evaporated by continuous stirring or by reducing the pressure, once the stable emulsion is formed. Several factors were found to affect the size range of nanoparticles. Among these factors are the concentrations and type of both the stabilizer and the polymer, and the homogenizer speed [58]. Ultrasonication or high-speed homogenization may be used to fabricate small particle size. Ultracentrifugation is used to collect nanoparticles, then they are washed using distilled water to remove stabilizer residue or any free drug. Nanoparticles are further lyophilized for storage. There are two modifications for this method; solvent evaporation method and high pressure emulsification [59]. The latter method involves preparation of an emulsion which was homogenized under high pressure followed by removal of organic solvent by steering. Many drugs activity were improved and enhanced by preparation as nano-formulation using solvent evaporation method, examples include improved skin penetration of ibuprofen [60] and Betulinic acid nanoparticles as Visceral Leishmaniasis alternative treatment [61].

##### Spontaneous emulsification

This method works with both lipophilic and lipophobic medicines. A multiple w/o/w emulsion with the medication dispersed in the internal aqueous phase is required for lipophobic drugs [62].

##### Double emulsion and evaporation method

Many evaporation-based techniques have the disadvantage of poor hydrophilic drug entrapment. To load the lipophobic drug, the double emulsion approach is employed, by adding drug solutions to an organic solution, containing the polymer, while continuously stirring to produce a w/o emulsion. The generated emulsion is then continuously incorporated into the second aqueous phase. To make the w/o/w emulsion, keep swirling. The solvent is then evaporated, and the nanoparticles may be separated using high-speed centrifugation. Before lyophilization, the produced nanoparticles must be cleaned. The quantity of integrated hydrophilic drug, the stabilizer concentration, the polymer concentration, and the volume of aqueous phase all have a role in the characterization of nanoparticles in this procedure [63]. Some examples for drugs nano-formulations prepared by double emulsion technique include Rose Bengal for breast cancer treatment [64] and oleuropein with improved stability [65].

##### Salting out method

Salting-out effect depends mainly on the separation of a water miscible solvent from aqueous solution. Both the medication and the polymer are dissolved in a vehicle in the first phase, which is then emulsified into an aqueous gel with the salting out agent and a colloidal stabilizer. Salting out agents (electrolytes, as well as non-electrolytes) and colloidal stabilizers have been used [66]. This technique produces an oil/water emulsion, which is subsequently diluted with enough water to enhance solvent diffusion in the aqueous phase, allowing for the formation of nanospheres. Salting out technique is used for the synthesis of ethyl cellulose, PLA and Poly (methacrylic) acids nanospheres. This method has the advantage of minimizing the stress on the protein included in encapsulants formation and resulted in high efficiency and is easily scaled up [67].

##### Emulsions–diffusion method

Another way to make nanoparticles is to use this technique. To establish the first thermodynamic equilibrium of both liquids, the encapsulating polymer is dissolved in a partly water-miscible solvent (such as propylene carbonate or benzyl alcohol) and saturated with water. The polymer-water saturated solvent phase is then emulsified in an aqueous solution containing a stabilizer, causing solvent diffusion to the exterior phase and the production of nanospheres or nanocapsules, depending on the oil-to-polymer ratio. In the last step, the solvent is removed by evaporation or filtration, depending on its boiling point. This technique has several benefits, such as high encapsulation efficiencies, no need for homogenization, high batch-to-batch reproducibility, scale up easily, simplicity, and narrow size distribution [44]. This technique was used for preparation of estrogen-loaded PLGA-nanoparticles [58] and synthesis of poly lactic acid [68].

##### Solvent displacement method

In this process, pre-formulated polymer is precipitated in an organic solution, while the organic solvent is dispersed in the aqueous solution [69]. Surfactants can be added to aid the diffusion of organic solvent [70]. After completely dissolving, the solution is injected into an aqueous solution containing a stabilizer while being constantly stirred. Fast solvent diffusion causes nanoparticles to develop spontaneously. The solvent is then removed from the suspensions at low pressure after this phase. The ratio of the organic phase to the aqueous phase determines particle size. It is well understood that raising the mixing rate of the two phases reduces particle size and drug entrapment [69]. This technique is not suitable for encapsulating of hydrophilic drugs but limited for those with poor solubility with an advantage of the ease of scaling up for industrial production [6]. Regulating the polymer concentration in the organic phase has proven to be effective in the formulation of smaller sized nanospheres. Nonetheless, size range is confined to minimum range of the polymer to drug ratio [44]. Examples for preparations were done by this technique include preparation of Boldine-loaded PLGA nanoparticles [71], Functionalized polyaniline nanoparticles [72], N-Acetylcysteine loaded in PLGA nanoparticles [73] and Non-isocyanate polyurethane nanoparticles [74].

#### Coacervation or ionic gelation method

Ionic gelation could be utilized for the production of hydrophilic polymer based nanoparticles [75]. This technique is first reported by Calvo and coworkers in 1997 [76, 77]. It depends on the great electrostatic attraction between positively charged amino group of chitosan as well as negatively charged tripolyphosphate where two different aqueous phases were prepared, one for polymer and the other is for polyanion sodium tripolyphosphate to formulate coacervates that have a size in nanometer range [78]. Other examples used this technique involve the preparation of advanced controlled released chitosan nanoparticles with improved properties of less aggregation tendency using tripolyphosphate-beta-cyclodextrin complex [79], levofloxacin loaded polymeric nanoparticles [80], and encapsulation of anthocyanins antioxidant for improved stability [81].

#### Polymerization method

This method is done in aqueous solution by polymerization of monomers to form nanoparticles. Drug is incorporated by two different methods during polymerization process (either by diffusion in the polymerization medium or by adsorption onto the nanoparticles after complete polymerization) [82]. Ultracentrifugation can be utilized to separate nanoparticle suspension from different stabilizers and surfactants that were used during polymerization, followed by the re-dispersion of the nanoparticles in an isotonic medium free from surfactants. The desired size of nanoparticles can be obtained by optimizing concentration of the surfactants and stabilizers. There are many applications and researches were achieved using polymerization technique include synthesis of super hydrophobic cotton fabrics [83, 84] and fabrication of nonporous polyimide silsesquioxane nanostructure as soft dielectric materials [85].

#### Nano spray drying

Spray drying is a fast, simple, reproducible, and scalable drying technology, which allows mild temperature condition suitable for heat-sensitive biopharmaceutical compounds. Spray drying, in comparison to other drying methods, is a continuous process that converts different liquids to solid particles while allowing for size, distribution, shape, porosity, density, and chemical composition adjustments. Spray drying equipment are commercially accessible, and the cost of manufacturing is cheaper than other drying methods such as freeze drying [86]. Spray drying involves four steps: (1) heating the drying gas, (2) droplet production, (3) droplet drying, and (4) particle collecting. Nano spray drying enables the generation of smaller particle sizes than conventional spray dryers that improves bioavailability and release of bioactive components and drugs. Drug-loaded nanoparticles provide several benefits, including a greater surface-to-volume ratio, a better rate of cell penetration, increased stability, and the capacity to pinpoint release [87].

#### Supercritical fluid technology

The previously mentioned conventional methods utilize organic solvents that are dangerous to the environment and the physiological systems. Supercritical fluid technology has been utilized as an alternative to manufacture biodegradable micro- and nanoparticles since supercritical fluids are ecologically friendly [88]. Even though environmentally friendly and suitability for mass production, supercritical fluid technology needs specific expensive equipment. Supercritical fluids are fluids, when are at a temperature higher than its critical temperature, still remain homogenous, regardless of pressure. Supercritical CO_2_ (SC-CO_2_) is the most broadly applied supercritical fluid due to its moderate critical conditions, non-flammability, considerable price and safety [89].

### Applications of nanoparticles

#### Nanoparticles in the treatment of kidney diseases

In urology and nephrology, nanoparticles are utilized to treat renal disorders. Ferumoxytol has been included into nanoparticles for the treatment of patients with chronic kidney disease or end-stage renal disease who do not produce enough erythropoietin [90]. Due to the onset of numerous diseases from this area, PEGylated gold nanoparticles can also target the mesangium—contractile cells that make up the central stalk of the glomerulus of the kidney. Rhein, an anthraquinone derivative used to treat diabetic nephropathy, had its distribution and therapeutic impact increased thanks to nanoparticle technology. Triblock amphiphilic was used to make Rhein nanoparticles. Rhein nanoparticles were synthesized using triblock amphiphilic polymers, namely polyethylene glycol-co-polycaprolactone-co-polyethylenimine. The size of nanoparticles prepared was about 75 nm which is optimum for kidney-targeted drug delivery. The results demonstrated that distribution to kidney as well as therapeutic effects of the drug were improved [83].

#### Nanoparticles for treatment of tuberculosis by chemotherapy

The improved efficacy of the anti-TB medicines loaded nanoparticles was due to their changed release behavior following oral administration. Rifampin, isoniazid, and pyrazinamide, three major medicines were co-incorporated in PLG-nanoparticles. These medications' therapeutic concentrations in tissues were kept for 10 days, but free drugs only lasted 1 day in the plasma after injection [91, 92].

#### Nanoparticles topical drug delivery for skin diseases

PNPs are the most extensively used nanoparticles for medication administration on the skin [93]. PNPs made from chitosan and alginate are used to treat acne, and as compared to benzoyl peroxide alone, they showed improved antibacterial efficacy against Propionibacterium acnes [94, 95]. In addition to polymeric nanoparticles, electro-spun fibres mats have a high surface area-to-volume ratio, which helps with the effective dispersion of both hydrophobic and hydrophilic medications and making them ideal for topical drug administration [93, 96]. Liposomes, solid lipid nanoparticles (SLN), and nanostructured lipid carriers (NLC) all cling to the surface of the skin. Liposomes, solid lipid nanoparticles (SLN), and nanostructured lipid carriers (NLC) bind to the skin's surface, allowing lipid exchange between the stratum corneum's outermost layers and the carrier for better medication penetration. Inflammatory skin illnesses including psoriasis and atopic eczema were treated with lipid-based carrier systems containing glucocorticoids and T-cell suppressing drugs like cyclosporin and tacrolimus. Surface modified SLNs containing retinyl palmitate improved the drug's cutaneous dispersion when compared to neutral SLNs, according to Jeon et al. [97]. Recent research has revealed that incorporating retinol into Compritol-based SLN make the drug released more rapidly as compared to conventional carrier [98–100].

#### Drug targeting to infectious diseases by nanoparticles

The physical and chemical properties of nanoparticles are being used as a tool to treat a variety of infectious disorders. The use of a therapeutic medicine loaded on a nano-vector has boosted the efficacy of the drug against infectious disorders. The polyethylene glycol-modified carbon nanotubes are the most common type of non-viral delivery system due to their enhanced pharmacokinetic and toxic characteristics. These are efficient carriers of bioactive molecules in the delivery of specific drugs for the treatment of infectious illnesses [101, 102].

#### Applications of nanoparticles in treating Alzheimer’s disease

Nanoparticle-mediated medication delivery is one of the newest approaches for increasing CNS penetration for the diagnosis and treatment of neurodegenerative diseases like Alzheimer's disease. PNPs are promising candidates among the various nanocarriers used because, in addition to being able to open the tight junctions of the Blood Brain Barrier, they effectively conceal the membrane barrier confining characterizations of the drug molecule, prolonging drug release and protecting drugs against enzymatic hydrolysis [103, 104].

#### Nanoparticles containing different anticancer agents

Nano-oncology is a new discipline of medicine that makes use of nanoparticles to treat cancer. The use of nanoparticles as an effective medication improves cancer cell targeting and overcomes cancer tissue multidrug resistance [105]. PLGA is a widely used polymer for making nanoparticles, and it has been utilized to make drug-loaded nanoparticles for cancer therapy due to its biocompatibility and long-term drug release. PLGA has been used to successfully manufacture anticancer drugs such as doxorubicin, 5-fluorouracil, paclitaxel, and dexamethasone. The FDA authorized Nutropin Depot, a microsphere version of Somatropin-PLGA nanoparticle, in 1999 as a once-time treatment. Nutropin Depot, a microsphere version of Somatropin-PLGA nanoparticle, was approved by the FDA in 1999 as a once-per-month alternative to daily HGH injections [106]. Doxorubicin is an anticancer medication that is primarily used to treat a variety of cancers. This feature limits its therapeutic potential because it is a very toxic substance that affects not just tumour tissue but also the heart and kidney. The creation of doxorubicin in liposomes, on the other hand, resulted in an FDA-approved nanomedical drug delivery system [107]. This new liposomal formulation lowered doxorubicin transport to the heart and kidney while increasing doxorubicin accumulation [108].

#### Nanoparticles in vaccination against COVID-19

During the year 2020 and now, all scientists and researchers are concentrating their efforts on creating remedies to combat the worldwide epidemic of the COVID-19 virus. In the year 2021, the importance of nanoparticle technology in the development of therapeutic formulations for the diagnosis, treatment, and promotion of long-term human immunity against COVID-19 was highlighted [109]. The backbone in succession and acceleration of the time required for creation of COVID-19 nanoparticle-based vaccines (CNPBV) was the recorded genome structure from Corona viruses and the pre-knowledge of the sequence of the protein laying the virus surface [110]. Spikes are present.

The presence of spike proteins on the outer surface of the COVID-19 virus, which have a high connecting tendency toward nano-formulations and a high binding tendency toward host cell receptors, was employed as a key characteristic in the development of CNPBV [111]. A promising vaccine based on nanotechnology was approved by the food and drug administration (FDA) and proved its big value in prophylaxis against COVID-19 virus with a high percentage of 90% on the vaccinated population among various vaccines produced with moderate efficacy to fight and limit the spread of the COVID-19 pandemic around the world. Pfizer-BioNTech (BNT162b2 vaccine) and Moderna vaccine (mRNA-1273 vaccine) are two of these vaccinations [112]. Pfizer-BioNTech (BNT162b2 vaccine) and Moderna vaccine (mRNA-1273 vaccine) are two vaccines that rely on mRNA to encode the COVID-19 virus's spike glycoprotein (S) and then incorporating the modified mRNA (which encodes the virus glycolprotein) into lipid-based nanoparticles [113]. The encapsulated modified mRNA then aids in the transport of the protein antigen (spike protein) to immune cells, stimulating T cell activity and inducing antibody immunological responses within the human body [114].

### FDA approved nanomedicines

In the last few decades, different nano-pharmaceuticals have been approved for clinical use from the food and drug administration (FDA). Among approved nano-drugs, liposomal, polymeric and micelles were represented and administered using oral, intravenous and transdermal routes. Table 1 [115] shows representative examples of FDA approved nano-medicines.

**Table 1:** Examples of FDA-approved nanomedicines

Trade name	Material description	Advantages	Indications	Approval year
Estrasorb™	Micellar Estradiol	Controlled delivery of the drug	Menopausal therapy	2003
Marqibo®	Liposomal Vincristine	Increased delivery to tumor tissue; decreased systemic toxicity resulting from side-effects	Acute lymphoblastic leukemia	2012
Onivyde®	Liposomal Irinotecan		Pancreatic cancer	2015
BNT162b2 vaccine (developed by BioNTech and Pfizer)	Nucleoside modified mRNA encoding the viral spike glycoprotein of SARS-CoV-2, encapsulated in lipid nanoparticles	Protection of the non-replicating RNA from degradation and allow it to be delivered into host cells after intramuscular injection	Prevention of coronavirus disease	2020
mRNA-1273 vaccine (developed by Moderna)	Lipid nanoparticle-encapsulated mRNA-based vaccine, which encodes the spike protein (S protein) of SARS-CoV-2	Lipid nanoparticles are playing a key role in protecting and transporting the mRNA effectively to the right place in cells	Prevention of coronavirus disease	2020

## Conclusions

Nanotechnology is a promising science with variety of advantages and applications in the medical field. It overcame the problems associated with conventional drug delivery systems and took the chance to accomplishment in production of COVID-19 vaccines based on lipid nanoparticle with higher efficiency over the others conventional vaccines. More efforts are needed to increase the number of FDA approved nano-drugs and further studies must be done to understand the development of the unique properties of these magical particles.

## Data Availability

Not applicable.

## Abbreviations

API: Active pharmaceutical ingredient
SWNTs: Single-walled nanotubes
MWNTs: Multiwalled nanotubes
QDs: Quantum dots
PNPs: Polymeric nanoparticles
SLN: Solid lipid NPs
SEDDS: Self-emulsified drug delivery systems
o/w: Oil-in-water
NLC: Nanostructured lipid carriers
CNPBV: Nanoparticle-based vaccines
